# Left ventricular and atrial strain and the risk of mortality and rehospitalization in heart failure

**DOI:** 10.1186/s44156-026-00106-6

**Published:** 2026-02-16

**Authors:** Haris Zilic, Hannes Holm, Linda S. Johnson, Amra Jujic, Martin Magnusson

**Affiliations:** 1 Department of Clinical Physiology, Lund University, Malmö, Sweden; 2 Department of Cardiology, Lund University, Malmö, Sweden; 3 Department of Clinical Sciences, Lund University, Malmö, Sweden; 4 Wallenberg Centre for Molecular Medicine, Lund University, Malmö, Sweden; 5 Hypertension in Africa Research Team (HART), NorthWest University Potchefstroom, Potchefstroom, South Africa

**Keywords:** Heart failure, Global longitudinal strain, Left atrial strain, Mortality, Hospitalization

## Abstract

**Background:**

Global longitudinal strain (GLS) and left atrial strain metrics, including reservoir (LAr), contraction (LAct), and conduit strain (LAcd), have emerged as key indicators of left ventricular (LV) function and filling pressures. However, the prognostic value of these markers for risk stratification in acute heart failure (HF) remains uncertain, particularly in identifying patients at elevated risk of rehospitalization and mortality.

**Results:**

In the prospective HARVEST cohort study, LA strain and GLS measurements were obtained retrospectively in 141 patients (mean age 71 ± 13, 25% women). Strain values are reported as absolute values reflecting the magnitude of deformation regardless of sign. Multivariable adjusted Cox regression was used to test whether GLS, LAr, LAct, and LAcd were associated with all-cause mortality and HF rehospitalization. Hazard ratios were calculated per 1% decrease in strain values. During a median follow-up time of 39 (IQR 14–66) months (490 patient-years) for mortality analyses and 22 (IQR 4–51) months (354 patient-years) for HF rehospitalization 62 (44%) patients died, and 62 (44%) were rehospitalized. Higher GLS, LAr, and LAcd were associated with a lower risk of mortality (HR:0.94, 95%CI:0.89–0.99, *p* = 0.045; HR:0.93, 95%CI:0.89–0.98, *p* = 0.009; and HR:0.94, 95%CI:0.88–0.99, *p* = 0.039, respectively), and higher LAr and LAct were associated with reduced risk of HF rehospitalization (HR:0.93, 95%CI:0.88–0.98, *p* = 0.004; and HR:0.85, 95%CI:0.77–0.94, *p* = 0.002, respectively).

**Conclusion:**

In patients with acute HF, strain parameters predict prognosis, with poorer outcomes. Notably, decreasing LAr was associated with increased risk of both death and rehospitalization for HF, whereas decreasing GLS was only associated with higher mortality risk.

**Supplementary Information:**

The online version contains supplementary material available at 10.1186/s44156-026-00106-6.

## Introduction

Heart failure (HF) is a leading cause of morbidity and mortality worldwide, characterized by an impaired cardiac ability to maintain adequate blood perfusion due to structural and/or functional cardiac abnormalities [1, 2]. Hence, identifying prognostic non-invasive markers associated with mortality and rehospitalization may improve patient outcomes. Quantification of left ventricular ejection fraction (LVEF) is the primary measurement used in clinical practice to assess LV function, stratify HF severity, guide treatment decisions and predict prognosis [3, 4]. However, LVEF measurements are load-dependent, have limited accuracy in assessing LV function across varying heart rates, and do not differentiate between distinct HF aetiologies [2, 5, 6]. Furthermore, under the course of the patients HF aliment they may have different trajectories that transitions them from one category to another [4]. For this reason, novel prognostic markers in HF are needed.

A growing number of studies have suggested that global longitudinal strain (GLS) and left atrial (LA) strain may be useful for diagnosis and prognosis in HF, including to assess risk of all-cause mortality and HF hospitalization [4, 7, 8]. In comparison to LVEF, GLS is more sensitive in detecting subclinical LV dysfunction, less influenced by changes in preload and afterload, and reflects LV function more accurately across a range of heart rates [9, 10]. In the progression of HF, the LA strain metrics such as LA reservoir (LAr), LA conduit (LAcd) and LA contractile (LAct) have been implicated in the progression of LV dysfunction [7, 11–14], and are recommended by the American Society of Echocardiography (ASE), the European Association of Cardiovascular Imaging (EACVI) and the British Society of Echocardiography (BSE) to evaluate elevated LV filling pressures in HF [13, 15]. Given that the LA acts as a critical interface between the LV and the pulmonary circulation, its functional decline directly increases right ventricular (RV) afterload. Once the compensatory capacity of the LA is compromised, pressure and volume overload precipitate pulmonary venous congestion, impair gas exchange, and promote vascular remodelling. Thus, LA strain metrics may be particularly valuable for monitoring patients with acutely decompensated HF, where early detection of LA dysfunction could guide timely clinical interventions [16].

While there has been an increase of studies evaluating the prognostic value of GLS and LA strain parameters in patient hospitalized for acute HF [2, 7–9], comprehensive comparative analyses of different strain metrics for outcome prediction remain limited. Here, we evaluate the association of GLS and key LA strain components with all-cause mortality and HF rehospitalizations in patients with acute HF.

## Methods

### Study population

**Fig. 1:**
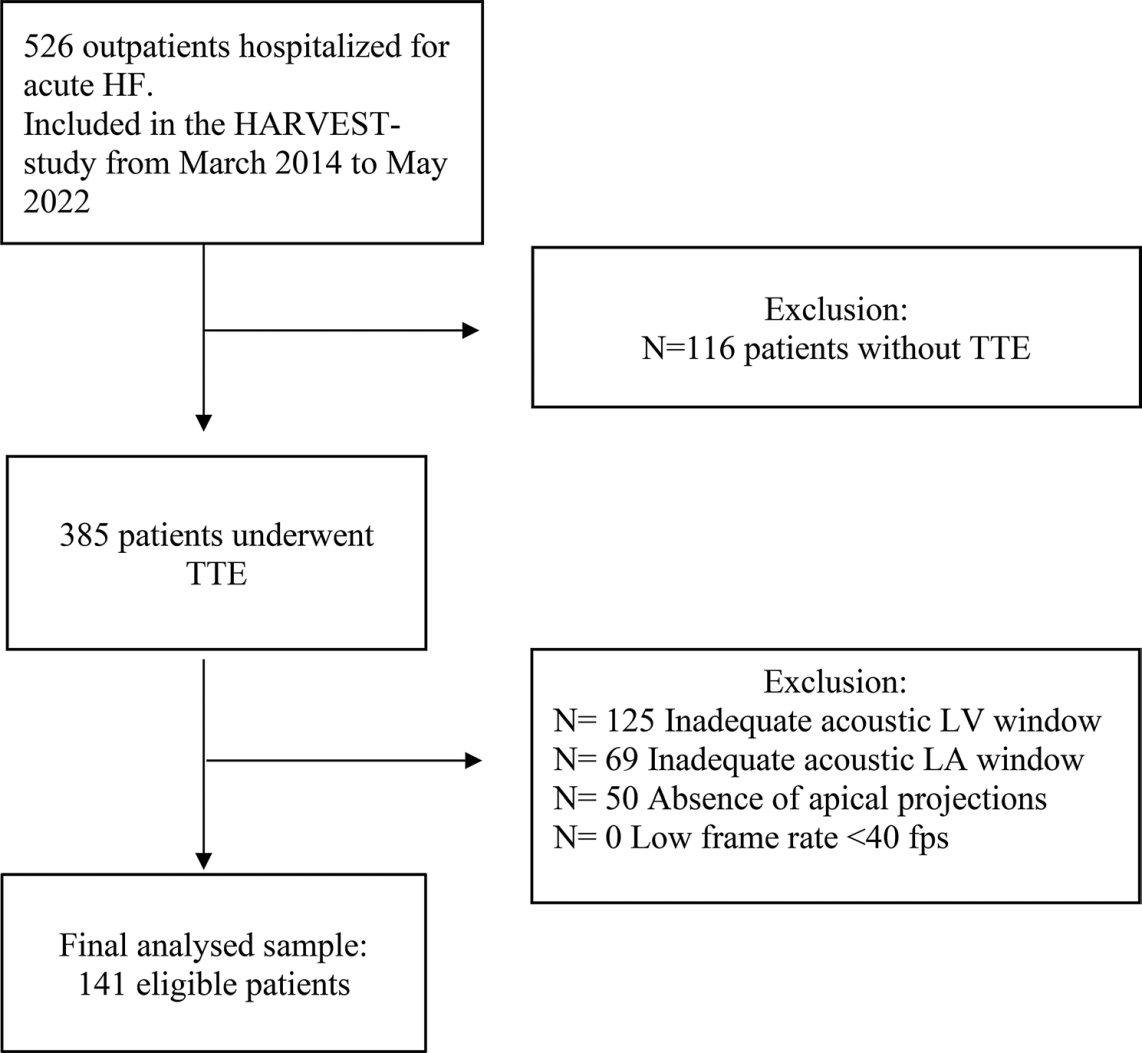
Flow chart of patient selection. HF, heart failure; HARVEST, The Heart and Brain Failure investigation; LA, left atrium; LV, left ventricle; TTE, transthoracic echocardiography

We conducted a retrospective analysis within the ongoing, prospective HARVEST-Malmö cohort study (The Heart and Brain Failure investigation) which includes 526 patients hospitalized for newly diagnosed acute or acute on chronic HF, at the Skåne University Hospital in Malmö, Sweden [17]. The inclusion of patients took place between March 2014 and May 2022. Among these, 385 participants underwent transthoracic echocardiograms (TTE), in whom the image quality for speckle tracking was sufficient in 141 patients, Fig. 1. The current sub study was not predefined at the time of initial population recruitment and was designed retrospectively based on evolved interest in strain metrics and their potential prognostic value. The study was approved by the Ethical Review Board at Lund University, Sweden and complies with the Declaration of Helsinki. Patients and/or the public were not involved in the design, or conduct, or reporting or dissemination plans of this research.

### Clinical assessment

Upon hospitalization and subsequent admission to the clinical wards, study participants underwent anthropometric measurements and had blood samples drawn after an overnight fast. Body mass index (BMI) was calculated as kilograms per square meter, and medication use was collected by self-report. Diabetes prevalence was defined as a self-reported diagnosis of type 2 diabetes, the use of antidiabetic medication, or a fasting plasma glucose > 7 mmol/L. Smoking status was self-reported as either ever or never-smokers. Trained nurses measured blood pressure (BP) using a validated automated BP monitor (Boso Medicus, Bosch + Sohn GmbH u. Co. KG, Jungingen, Germany). The appropriately sized upper arm cuff was placed on the right arm, supported at heart level. Hypertension was defined as systolic BP (SBP) ≥ 140 mmHg and/or diastolic BP (DBP) ≥ 90 mmHg. Atrial fibrillation (AF) was identified either by its presence on an electrocardiogram at the time of hospitalization or by a documented history of AF in the patient’s medical records. Kidney disease was considered prevalent if the estimated glomerular filtration rate (eGFR) was < 60 ml/min/1,73m^2^.

### Laboratory assays

Fasting plasma glucose was analysed using a Siemens Attelica at Department of Clinical Chemistry at Skåne University Hospital, Malmö, using standardized procedures. Measurements of total N-terminal pro brain natriuretic peptide (NT-proBNP), creatinine, and other biomarkers were carried out at the Department of Clinical Chemistry, Skåne University Hospital in Malmö. Plasma creatinine and cystatin C were analysed using Atellica^®^ CH analyzer. Relative eGFR was estimated as mean from creatinine based eGFR estimated using the Malmö-Lund revised formula and cystatin C based eGFR using the CAPA-formula (mL/min/1.73 m².) [18].

### Endpoints

Mortality was defined as death by any cause (total mortality) and was retrieved from the National Board of Health and Welfare’s Cause of Death Register, which includes all deaths that occur in Sweden. Data regarding rehospitalizations due to HF were retrieved from electronic medical charts which were available for all study subjects (Melior, Siemens Health Services, Solna, Sweden). All subjects were followed from study inclusion until Dec 31, 2022.

### Echocardiography

Participants in this study were all examined by experienced sonographers as a clinical routine at admission to cardiology or internal medicine wards at Skånes University Hospital Malmö, Sweden. Transthoracic echocardiography was performed with Philips IE33 (Philips, Andover, MA USA) with a 1–5 MHz transducer (S5-1), Philips Epiq (Philips, Andover, MA USA) with a 1-5 MHz transducer (S5-1/X5-1) or using the GE Vingmed Vivid 7 ultrasound system (GE, Vingmed Ultrasound, Horten Norway) with a 1–4 MHz transducer (M3S). The images were digitally stored in IntelliSpace Cardiovascular version 5.2.0.4 (Philips Medical Systems, Netherlands) and analysed offline.

LA strain and GLS were measured with speckle tracking and analysed offline with dedicated software (2D Cardiac Performance Analysis version 5.5.7 TomTec Imaging Systems, Unterschleissheim, Germany). A single expert operator (HZ) with prior specific training in LA and LV strain analysis and experience in measuring LA and LV strain performed all speckle tracking (strain) analyses. To assess the intra-observer reproducibility, a random sample of 15 echocardiograms was reanalysed by the same operator. The echocardiographic views used for performing and measuring the LA-strain were non-foreshortened apical four-chamber of the LA. A 3-click region of interest (ROI) was used to capture the left atrial myocardium, thereby defining endocardial borders (inner contour of the LA wall) on both sides of the mitral valve and the atrial roof. To ensure that the ROI was adequately placed some manual adjustments were made to fit the thickness of the atrial myocardium, thereby avoiding inclusion of the pericardium [19, 20]. The R-wave on the ECG defined by mitral valve closure (left ventricular end-diastole) was chosen as the baseline reference point (zero strain) [19]. The LA-strain parameters analysed were: LA reservoir phase (LAr), LA conduit phase (LAcd) and LA contraction phase (LAct). In patients with AF during the echocardiographic exam (*n* = 49, 35%), speckle-tracking analysis was performed using the R-wave on the ECG as the zero reference with LAr, LAcd and LAct quantified across multiple consecutive beats to account rhythm irregularities. Even though the software provided LAct values in patients with AF, their interpretation was considered exploratory given the absence of atrial contraction [20]. Atrial wall dilatation during the reservoir phase, should be expressed as a positive strain value, whereas the contraction of the LA wall during the other two phases should be characterized by negative values [20]. Both LA strain and LV strain values are reported as absolute values reflecting the magnitude of deformation regardless of sign. GLS measurements of LV were performed using non-foreshortened apical four-chamber (A4C), two-chamber (A2C), and three-chamber (A3C) views at the endocardial borders. For quantification of LV GLS, the AutoStrain application (2D Cardiac Performance Analysis version 5.5.7 TomTec Imaging Systems, Unterschleissheim, Germany) was used which utilizes two automation technologies: Auto View Recognition and Auto Contour Placement. The Auto View Recognition automatically identifies which selected image is A4C, A2C and A3C, and thereby assigns the labels to the selected images, whereas Auto Contour Placement is a specialized contour detection module. In some cases, manual adjustments were required. In images with poor tracking in two or more segments, LV strain was not measured. Patients were excluded from the study if they had inadequate acoustic window for the LV or LA, defined as more than two non-visible LV or LA segments, absence of apical view images and echocardiographic recordings with a framerate lower than 40 frames per second (fps) or did not have an echocardiographic exam within 72 h of admission.

### Statistics

Continuous variables are presented as means (± standard deviation, SD), medians (25th-75th percentiles) or numbers (%). Differences in covariates are reported across quartiles of strain values and tested using one-way ANOVA test for normally distributed continuous variables, Mann-Whitney U-test for continuous variables with non-normal distribution, and χ2 test for binary variables. Associations between levels of strain parameters, mortality and rehospitalization risk were analysed using multivariable Cox regression analysis adjusted in 3 models. Hazard ratios were calculated per 1% decrease in strain. *Model 1* was adjusted for age and sex; *Model 2* was further adjusted for moderate kidney disease and prevalent hypertension. In *Model 3*, current smoking, fasting plasma glucose (FPG) and prevalent AF was added to Model 2 covariates. The outcomes of interest were all-cause mortality, and HF rehospitalization. Kaplan–Meier survival curves were generated to visualize time-to-event data, and the log-rank test was used to compare survival distributions between groups. The competing risk of death for HF rehospitalization was modelled using Fine-Gray subdistribution hazard regression with death as a competing event (outcome coded 0 = censored, 1 = rehospitalization, 2 = death). Time was measured in months from index admission to first event or censoring. Each strain parameter (GLS, LAr, LAcd LAct) was entered in separate models and expressed per 1% decrease (implemented by reversing the sign of the strain variable, corresponding to less negative strain values, reflecting worse function). Models were adjusted for age, sex, moderate chronic kidney disease, hypertension, log-transformed fasting plasma glucose, current smoking, and atrial fibrillation. Results are reported as subdistribution hazard ratios (sHR) with 95% confidence intervals and two-sided p-values. For additional analyses, multivariable Cox regression models were fitted including all three LA strain components (reservoir, conduit, contraction) simultaneously, in order to assess their independent prognostic contribution. Analyses were conducted in R using cmprsk (version 2.2–12). All-cause mortality was analysed with standard Cox regression (no competing risk for an all-cause endpoint). Intra-observer reproducibility was quantified with the intraclass correlation coefficient ICC (3, 1) (two-way mixed-effects, absolute agreement, single measures), calculated using the ICC function from the *psych*package in R (version 4.3.2). A two-sided p-value < 0.05 was considered statistically significant.

## Results

### Baseline characteristics

The analysed cohort consisted of 141 patients, the mean age was 71 (± 13) years, and 75% were males. HF was new-onset in 39% of patients and 57% had heart failure with reduced ejection fraction (HFrEF) (< 40%), 19% had heart failure with mildly reduced ejection fraction (HFmrEF) (41–49%), and 29% had heart failure with preserved ejection fraction (HFpEF) (≥ 50%). Baseline characteristics across quartiles of LAr and LAct are reported in Tables 1 and 2. The lower quartiles of LAr and LAct were associated with lower SBP and a higher prevalence of AF. Hypertension was less common in the lower LAct quartiles, while higher NT-proBNP levels were observed in the lower quartiles of LAcd. Additionally, a higher proportion of males was noted in the decreasing quartiles of GLS (Table 1). All strain parameters except LAct showed a significant decline by a decrease in LVEF, Tables 1 and 2. During a median follow-up time of 39 (IQR 14–66) months (490 patient-years) for mortality analyses and 22 (IQR 4–51) months (354 patient-years) for HF rehospitalization 62 (44%) patients died, and 62 (44%) were rehospitalized, where the most frequent cause of death was HF (*n* = 15) followed by cardiac arrest (*n* = 7), cancer (*n* = 1), and stroke (*n* = 1). The remaining recorded deaths (*n* = 38) were due to non-cardiovascular causes. These data are presented in Supplementary Table 3. Out of the 62 patients (44%) who were re-hospitalized for HF, 67% died during the follow-up period.

**Table 1:** Baseline characteristics variables across quartiles of LAr and GLS

Baseline characteristic		Quartiles of LAr					Quartiles of GLS				
	All, *n* = 141	1 (*n* = 35)	2 (*n* = 35)	3 (*n* = 36)	4 (*n* = 35)	*p*	1 (*n* = 34)	2 (*n* = 37)	3 (*n* = 36)	4 (*n* = 34)	*p*
		2.2–8.3	8.4–11.2	11.3–15.8	16–40		1.8–6.8	6.9–9.2	9.3–13	13.3–24.4	
Age (years; (SD))	71 (13)	73 (13)	72 (13)	71 (13)	69 (12)	0.549	71 (14)	69 (12)	72 (14)	73 (12)	0.535
Sex (male n; (%))	106 (75)	29 (83)	26 (74)	27 (75)	24 (69)	0.585	29 (85)	33 (89)	24 (67)	20 (59)	**0.007**
Current smoking (n; (%))	18 (13)	6 (17)	5 (14)	3 (8)	4 (11)	0.714	3 (9)	7 (19)	7 (19)	1 (3)	0.106
BMI (kg/m^2^; (SD))	27 (6)	27 (7)	27 (5)	27 (5)	27 (6)	0.981	27 (7)	27 (4)	26 (6)	29 (5)	0.212
SBP (mmHg; (SD))	140 (31)	132 (25)	132 (29)	150 (29)	147 (33)	**0.019**	134 (22)	136 (29)	142 (36)	149 (36)	0.172
DBP (mmHg; (SD))	81 (17)	79 (13)	80 (13)	82 (18)	83 (24)	0.832	80 (13)	82 (17)	80 (20)	82 (19)	0.937
Diabetes (n; (%))	49 (35)	10 (29)	14 (40)	14 (39)	11 (31)	0.692	12 (35)	9 (24)	12 (33)	16 (47)	0.253
Prevalent AF (n; (%))	49 (35)	20 (57)	14 (40)	8 (22)	7 (20)	**0.003**	10 (29)	15 (41)	12 (33)	12 (35)	0.799
Prior myocardial infarction	50 (36)	10 (29)	15 (43)	16 (44)	9 (26)	0.252	14 (42)	15 (41)	13 (36)	8 (24)	0.358
Moderate kidney disease (n; (%))	100 (71)	26 (74)	27 (77)	25 (69)	22 (63)	0.574	24 (71)	25 (68)	27 (75)	24 (71)	0.920
eGFR (mL/min/1.73 m2; (SD))	48 (18)	46 (18)	46 (17)	49 (17)	53 (19)	0.266	48 (17)	51 (17)	46 (19)	49 (17)	0.684
Hypertension (n; (%))	62 (44)	14 (40)	11 (31)	21 (58)	16 (46)	0.138	15 (44)	17 (46)	14 (39)	16 (47)	0.904
Prior heart failure (n; (%))	86 (61)	24 (69)	25 (71)	25 (69)	12 (34)	**0.003**	24 (71)	25 (68)	21 (58)	16 (47)	0.182
NTproBNP (pmol/L; (SD))	7092 (7507)	9192 (8149)	7799(8872)	6613(7057)	4886 (5102)	0.108	9248 (8447)	7925 (7729)	6596 (6504)	4670 (6830)	0.075
Ejection fraction (%; (SD))	37 (15)	30 (12)	33 (13)	42 (15)	44 (14)	**< 0.001**	25 (8)	31 (10)	42 (12)	53 (12)	**< 0.001**

BMI, Body mass index; SBP, systolic blood pressure; DBP, diastolic blood pressure; AF, atrial fibrillation; eGFR, estimated glomerular filtration rate

All strain values are presented as absolute values (%)

Bold values indicate statistical significance (*p* < 0.05)

**Table 2:** Baseline characteristics variables across quartiles of LAct and LAcd

Baseline characteristic	Quartiles of LAct					Quartiles of LAcd				
	1 (*n* = 35)	2 (*n* = 35)	3 (*n* = 35)	4 (*n* = 36)	*p*	1 (*n* = 35)	2 (*n* = 36)	3 (*n* = 35)	4 (*n* = 36)	*p*
	0.2–1.7	1.8–3.3	3.4–6.6	6.7–28.9		1.0-4.8	4.9–7.2	7.4–10.3	10.4–35.3	
Age (years; (SD))	73 (11)	73 (15)	71 (11)	67 (13)	0.179	72 (12)	71 (15)	71 (12)	72 (12)	0.974
Sex (male n; (%))	24 (69)	29 (83)	28 (80)	25 (69)	0.392	26 (74)	28 (78)	28 (82)	24 (67)	0.479
Current smoking (n; (%))	7 (19)	1 (3)	6 (17)	4 (11)	0.143	6 (17)	6 (17)	3 (9)	3 (8)	0.531
BMI (kg/m^2^; (SD))	27 (6)	27 (7)	27 (4)	28 (5)	0.867	27 (6)	26 (5)	27 (5)	28 (6)	0.487
SBP (mmHg; (SD))	133 (22)	134 (33)	133 (29)	160 (33)	**< 0.001**	139 (29)	141 (35)	142 (31)	139 (31)	0.957
DBP (mmHg; (SD))	79 (11)	80 (15)	76 (14)	88 (24)	**0.014**	80 (14)	84 (24)	83 (13)	77 (14)	0.317
Diabetes (n; (%))	11 (31)	12 (34)	12 (34)	14 (39)	0.930	14 (40)	9 (25)	13 (38)	13 (36)	0.543
Prevalent AF (n; (%))	23 (66)	14 (40)	9 (26)	3 (8.3)	**< 0.001**	14 (40)	11 (31)	10 (29)	14 (39)	0.705
Prior myocardial infarction	8 (23)	10 (29)	18 (51)	14 (39)	0.075	17 (49)	11 (31)	12 (35)	10 (28)	0.285
Moderate kidney disease (n; (%))	26 (74)	26 (74)	22 (63)	26 (72)	0.678	27 (77)	26 (72)	24 (71)	23 (64)	0.670
eGFR (mL/min/1.73 m2; (SD))	49 (14)	46 (19)	50 (20)	49 (19)	0.867	45 (17)	48 (19)	45 (16)	51 (19)	0.390
Hypertension (n; (%))	12 (34)	16 (46)	9 (26)	25 (69)	**0.001**	16 (46)	16 (44)	17 (49)	13 (36)	0.694
Prior heart failure (n; (%))	21 (60)	22 (63)	24 (69)	19 (53)	0.587	28 (80)	20 (56)	23 (68)	15 (42)	**0.007**
NTproBNP (pmol/L; (SD))	6376 (5773)	7070 (7496)	6493 (7360)	8409 (7480)	0.662	10,080 (9265)	8490 (8148)	6283 (6299)	3700 (3935)	**0.002**
Ejection fraction (%; (SD))	37 (14)	35 (17)	39 (14)	40 (15)	0.564	31 (13)	35 (12)	36 (13)	48 (15)	**< 0.001**

BMI, Body mass index; SBP, systolic blood pressure; DBP, diastolic blood pressure; AF, atrial fibrillation; eGFR, estimated glomerular filtration rate

All strain values are presented as absolute values (%)

Bold values indicate statistical significance (*p* < 0.05)

### Association between strain parameters, mortality, and rehospitalization risk

Intraclass correlation coefficient was evaluated for 15 echocardiograms and demonstrated good reliability, ranging from 0.87 for GLS to 0.97 for LAct, indicating excellent measurement consistency for all measurements. The coefficient of variation (CoV) further supported reproducibility, with values of 14.3% for LAr, 20.5% for LAcd, 14.0% for LAct, and 11.8% for GLS.

**Fig. 2:**
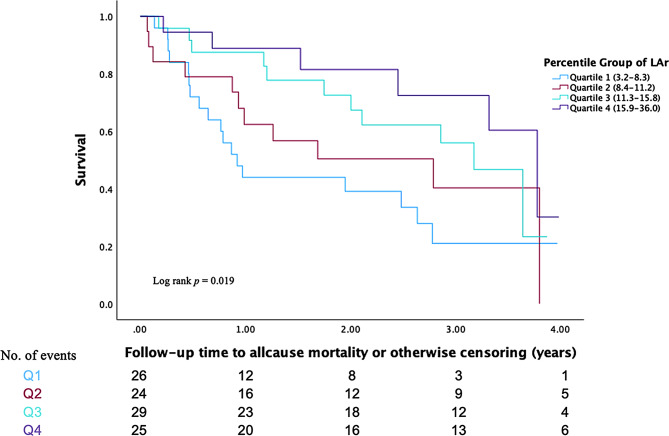
Kaplan Meier survival curves for the quartiles of LAr

**Fig. 3:**
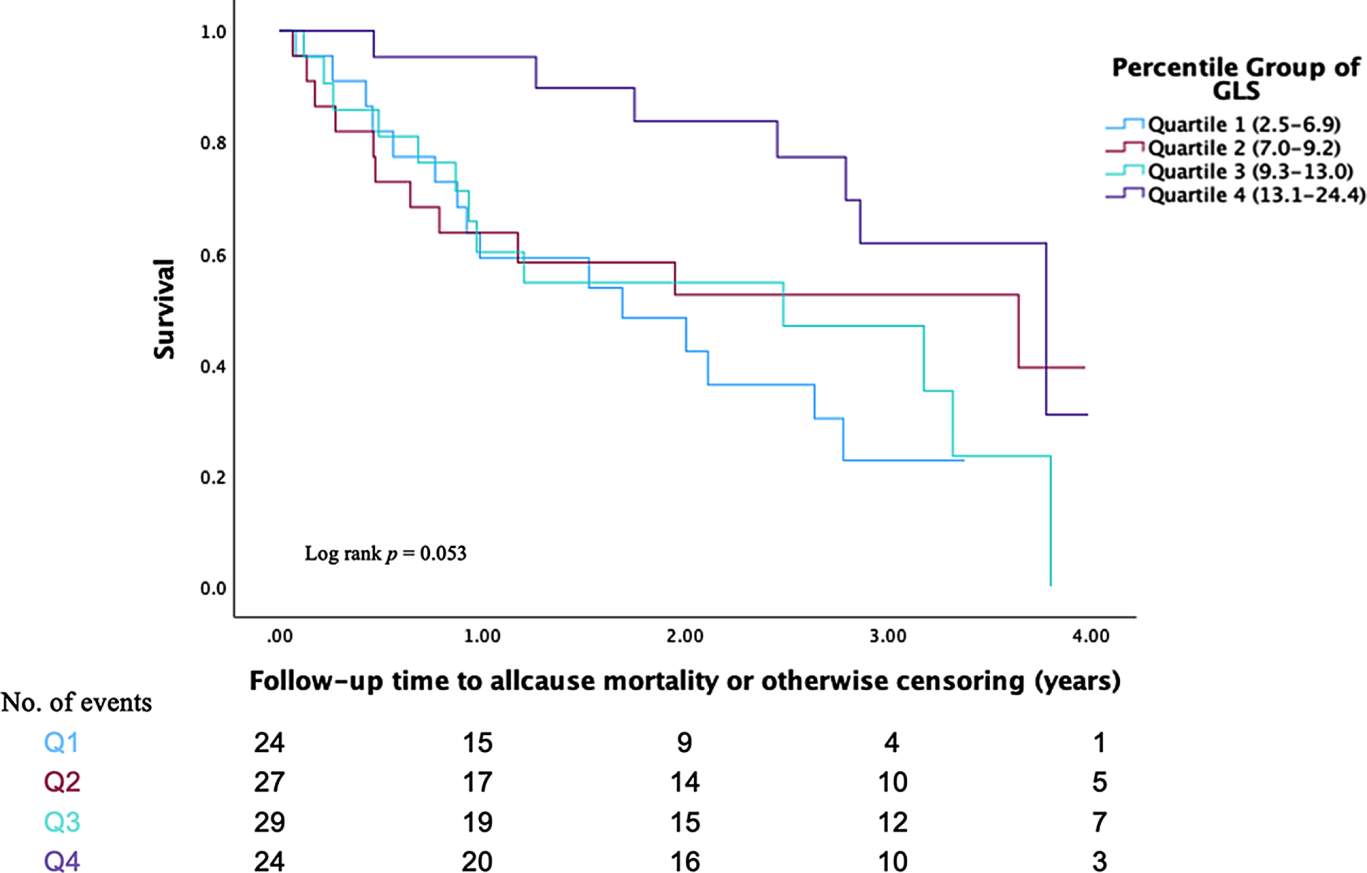
Kaplan Meier survival curves for the quartiles of GLS

Higher values of LAr, LAcd, and GLS were significantly associated with a lower risk of all-cause mortality, with hazard ratios (HR) of 0.93 (95% CI 0.89–0.98, *p* = 0.009), 0.94 (95% CI 0.88–0.99, *p* = 0.039), and 0.94 (95% CI 0.89–0.99, *p* = 0.045), respectively. These hazard ratios reflect the relative risk change per 1% decrease in strain. In the multivariate model, the only strain parameters associated with HF rehospitalization were LAr and LAct HRs of 0.93 (95% CI 0.88–0.98, *p* = 0.004) and 0.85 (95% CI 0.77–0.94, *p* = 0.002), respectively. Participants in the lowest quartile of LAr had an increased risk of all-cause mortality (HR 2.54; 95% CI 1.38–4.67, *p* = 0.003) but not HF rehospitalization (HR 1.57; 95% CI 0.84–2.95, *p* = 0.157). Conversely, participants within the lowest quartile of GLS had an increased risk of HF rehospitalization (HR 2.19; 95% CI 1.25–3.83, *p* = 0.006). However, the association between the lowest quartile of GLS values and all-cause mortality was not significant following adjustment, Table 3; Figs. 2 and 3. No significant association was observed between lower EF values and HF rehospitalization (HR 0.99; 95% CI 0.98–1.01; *p* = 0.283). However, lower EF values were significantly associated with an increased risk of all-cause mortality (HR 0.98; 95% CI 0.97–0.99; *p* < 0.001), supplementary Table 1.

**Table 3:** Cox regression analyses for the association between atrial strain parameters, GLS with all-cause mortality and HF rehospitalization

Variables	All-cause mortality		Heart failure rehospitalization	
	HR (CI 95%)	*p*-value	HR (CI 95%)	*p*-value
***Continuous LAr***				
*Model 1*	0.95 (0.91–0.99)	**0.020**	0.94 (0.90–0.98)	**0.006**
*Model 2*	0.95 (0.91–0.99)	**0.034**	0.94 (0.90–0.99)	**0.009**
*Model 3*	0.93 (0.89–0.98)	**0.009**	0.93 (0.88–0.98)	**0.004**
***Continuous GLS***				
*Model 1*	0.94 (0.87–0.99)	**0.024**	0.95 (0.90–1.01)	0.082
*Model 2*	0.94 (0.88–0.99)	**0.027**	0.95 (0.89–1.01)	0.066
*Model 3*	0.94 (0.89–0.99)	**0.045**	0.95 (0.90–1.01)	0.084
***Continuous LAct***				
*Model 1*	0.96 (0.90–1.03)	0.253	0.90 (0.83–0.98)	**0.013**
*Model 2*	0.97 (0.91–1.04)	0.426	0.91 (0.83–0.99)	**0.020**
*Model 3*	0.94 (0.87–1.03)	0.164	0.85 (0.77–0.94)	**0.002**
***Continuous LAcd***				
*Model 1*	0.94 (0.89–0.99)	**0.042**	0.95 (0.90–1.01)	0.078
*Model 2*	0.95 (0.89-1.00)	**0.050**	0.95 (0.90–1.01)	0.080
*Model 3*	0.94 (0.88–0.99)	**0.039**	0.95 (0.90–1.01)	0.112
***Lowest quartile of LAr***				
*Model 1*	1.78 (1.03–3.09)	**0.040**	1.44 (0.82–2.55)	0.209
*Model 2*	1.88 (1.08–3.27)	**0.025**	1.40 (0.79–2.49)	0.247
*Model 3*	2.54 (1.38–4.67)	**0.003**	1.57 (0.84–2.95)	0.157
***Lowest quartile of GLS***				
*Model 1*	1.65 (0.94–2.87)	0.079	2.13 (1.23–3.67)	**0.007**
*Model 2*	1.61 (0.91–2.84)	0.101	2.21 (1.27–3.84)	**0.005**
*Model 3*	1.52 (0.86–2.71)	0.150	2.19 (1.25–3.83)	**0.006**

Model 1: Adjusted for age and sex

Model 2: Adjusted for age, sex, moderate kidney disease, hypertension

Model 3: Adjusted for age, sex, moderate kidney disease, hypertension, fasting plasma glucose, current smoking, prevalent AF

Bold values indicate statistical significance (*p* < 0.05)

When including all three LA strain components (reservoir, conduit, and contraction) in the same Cox regression model adjusted for clinical covariates, only reservoir strain remained statistically significant in relation to all-cause mortality (HR 0.74; 95% CI 0.56–0.98, *p* = 0.035), while conduit and contraction strain did not (both *p* > 0.07). When all three LA strain components were analyzed simultaneously in relation to HF rehospitalization, none remained statistically significant, whereas moderate kidney disease and atrial fibrillation were independently associated with higher risk.

### Competing risk analyses

In Fine-Gray models adjusted for clinical covariates (63 HF rehospitalizations; 21 competing deaths), lower LAr and lower LASct were associated with a higher risk of HF rehospitalization (LAr: subhazard [sHR] 1.07 per 1% decrease; 95% CI 1.03–1.12; *p* = 0.0015; LAct: sHR 1.18 per 1% decrease; 95% CI 1.05–1.32; *p* = 0.0054). GLS and LAcd were not statistically significant (GLS: sHR 1.04, 95% CI 0.98–1.11, *p* = 0.16; LAcd: sHR 1.04; 95% CI 0.99–1.10; *p* = 0.10).

## Discussion

In this prospective long-term follow-up study in patients with acute HF, lower values of GLS and LA strain parameters were associated with poorer outcomes. Among the LA strain parameters, lower LAr was associated with higher risk of both death and rehospitalization for HF. Decreasing GLS was associated with higher mortality risk and patients in the lowest quartile had a significantly greater risk of HF rehospitalization. These outcomes show upon the possible prognostic importance of LA strain metrics, which reflect the compensatory capacity of the LA. Furthermore, they demonstrate that severe GLS impairment identifies patients at increased risk for rehospitalization. When accounting for the competing risk of death, impaired left-atrial reservoir and contraction strain remained independently associated with HF rehospitalization, underscoring the prognostic relevance of atrial mechanics beyond conventional measures. In line with prior studies, when all three LA strain components were considered simultaneously, only reservoir strain remained independently associated with adverse outcomes, underscoring its role as the most robust atrial strain marker in this setting. In our study, lower SBP was observed in patients with reduced atrial strain, which may reflect advanced decompensated heart failure where low cardiac output and vasodilatory states lower blood pressure despite long-standing hypertension and associated remodelling. While reservoir strain emerged as the most robust predictor of mortality, none of the atrial strain components remained significant for HF rehospitalization when considered together, highlighting the multifactorial nature of rehospitalization risk in this population. Our findings support the clinical relevance of combining GLS and LA strain to capture subtle impairments in LV function and filling pressures, which may be particularly useful in acute settings where prognostication must be made independently of preload, EF, or aetiology. The prognostic value of GLS differed depending on whether it was analyzed as a continuous variable or categorized into quartiles. This likely reflects loss of information and reduced power when converting a continuous variable into groups, particularly in a relatively small cohort. In the Kaplan-Meier analyses, the apparent convergence of survival curves beyond four years should be interpreted with caution, as only a few patients remained at risk in each quartile at that time, leading to unstable estimates.

While previous research has established LV and LA strain as significant predictors of adverse outcomes in HF patients, including both stable outpatients and patients hospitalized for acute HF [2, 7–9, 21], important gaps remain. Most existing studies have focused on LAr alone and predominantly excluded patients with AF, who encompass a great portion of acute HF admissions. Our study builds upon prior evidence in acute HF population [7–9] and extends this understanding by evaluating not only GLS and LAr but a broader set of LA strain parameters, including LAcd and LAct, as well as including a substantial number of patients with AF.

Consistent with the findings by Park et al. our study supports the notion of GLS as an independent predictor of mortality, despite differences in sample size (141 vs. 4172 participants) and cohort characteristics [9]. We also report on the association between LA strain measurements and adverse outcomes in patients acutely hospitalized for HF patients, who are often underrepresented cohort in studies examining strain metrics. Our observations align with a previous study of 85 consecutive acute HF patients, which demonstrated that LA dynamics were strong predictors of rehospitalization and cardiovascular outcomes [7].

In clinical settings, LVEF remains as the primary measurement for assessing LV function even though the use of serial echocardiographic measurements during follow-up to track LVEF over time is preferred over the use of single echocardiographic measurement [3, 4]. Our study provides further insights in which GLS may offer a more robust prognostic value beyond LVEF as it reflects myocardial contractility and thus is less affected by loading conditions [10]. Both our findings and those of Park et al. demonstrate the GLS values generally decrease with lower LVEF [9]. This is especially useful in cases where compensatory mechanisms can maintain LVEF within the normal range despite underlying myocardial dysfunction [9].

A growing number of studies highlights the prognostic value of LA strain in predicting adverse outcomes. In the current study, lower LAr was associated with both increased mortality and rehospitalization. Patients in the lowest quartile of LAr (< 8.3%) showed a significantly higher risk of all-cause mortality. This finding is consistent with previous studies suggesting that LA strain, specifically LAr serve as sensitive marker of LA compliance and reflects the hemodynamic burden imposed by elevated LV filling pressures [13, 21, 22]. Our study extends this understanding to acutely hospitalized HF patients, offering new insights into the prognostic value of these strain parameters and highlighting their relevance in acute care settings.

While earlier studies have shown strong correlations between LAr and invasive LV filling pressure, as well as NT-proBNP levels [2, 13, 22], our study supports this relationship by demonstrating a trend towards higher NT-proBNP levels in acute HF setting. Unlike conventional echocardiographic parameters such as left atrial index (LAVi) or E/é ratio which are less reliable in patients with LVEF < 50%, LAr remains unaltered even in the presence of AF or mitral valve disease [13, 21, 23]. Although our study did not directly examine LV filling pressure, based on the LVEF, LAr, LAct and GLS parameters, we reason that our patient group likely had elevated filling pressure [2, 23].

### LA strain and AF

Importantly, 35% of our cohort had AF, a subgroup often excluded from strain research [8]. This represents a clinically relevant population that reflects the real-world population of patients hospitalized for HF. Considering the high prevalence of AF reported in HF (23–65%), it is crucial to develop reliable assessment methods for LA function in this population, as impaired LA strain may be a key indicator of advanced atrial pathology [24]. We observed significantly lower LAr and LAct values in patients with AF. This is not unexpected, given that AF induces atrial desynchrony and atrial remodelling. While prior studies have found LAr less predictive in AF [8], our inclusion of LAct and LAcd offers a more nuanced view of atrial function. It is important to note that in patients with AF, the atrial contraction is absent. We acknowledge the limitations in LAct interpretation, but excluding patients with AF would have substantially reduced the generalizability of our findings given their high prevalence in HF. Even so, reduced LAct value may still reflect the degree of structural and functional atrial remodelling. Although LAr and LAct are related, they reflect different aspects of atrial mechanics and evaluating LAct in AF populations could thus offer valuable prognostic insights, complementing existing measures like LAr. Further studies in larger, rhythm-stratified populations are needed to fully explore these relationships.

We acknowledge that LA conduit strain (LAcd) is often reported as a negative value, where more negative values typically reflect better conduit function. However, in our dataset, LAcd was expressed in absolute terms, as is standard in several software packages and publications. Thus, in our analysis, higher LAcd values represent better conduit function and are logically associated with lower mortality risk. This should be clarified to avoid misinterpretation. Although our primary endpoint was all-cause mortality, most events were non-cardiac, with only 21 of 62 deaths attributed to heart failure or cardiac arrest. While strain parameters may plausibly predict cardiac-specific mortality, an association with non-cardiac death is less expected and may reflect shared underlying risk factors or frailty. This limits the specificity of our findings and underscores the need for future studies with adjudicated cardiac endpoints.

### Clinical implications

In clinical practice, implementing GLS and LA strain into routine echocardiographic assessment of patients hospitalized for HF, may provide incremental information on adverse remodelling and heightened risk beyond conventional measures. For instance, patients with severely impaired LAr exhibited a significant higher risk of all-cause mortality suggesting that LAr could serve as a critical marker for prioritizing high-risk patients for intensified monitoring or specialized treatment from the time of admission. Similarly, patients with severely impaired GLS were linked to significantly higher risk of HF rehospitalizations, suggesting that GLS could serve as a valuable tool to guide decisions on follow-up frequency and optimization of medical treatment. Together these strain measurements could complement conventional parameters thereby supporting early risk stratification, closer monitoring, and optimization of medical treatment.

### Limitations

This study has several limitations. First, although HARVEST-Malmö is a prospective study, the strain analysis was performed retrospectively using echocardiographic images that were not optimized for speckle tracking. The echocardiographic images were obtained during routine clinical care in an acute setting, often in hemodynamically unstable patients. As a result, the image quality was frequently suboptimal, and strain measurements were feasible in 141 of the 385 (37%) patients who underwent TTE. This relatively low proportion highlights the challenges of acquiring high-quality images in acute HF and should be considered when interpreting the generalizability of our findings. Although echocardiography was part of routine clinical care, participants in the current study were only included if the examination had been performed and was available for analysis. In some cases, echocardiography was not conducted due to early discharge, clinical instability, or logistical reasons. Further, echocardiographic examinations were performed as part of routine clinical care, and all patients were included within 72 h of admission. The timing could therefore vary depending on logistical factors such as staff availability during weekends. We acknowledge that some patients may have received initial treatment before the examination, which could potentially influence strain measurements. Also, in the Kaplan–Meier analyses, the apparent convergence of survival curves beyond four years should be interpreted with caution, as only a few patients remained at risk in each quartile at that time, leading to unstable estimates. We did not adjust strain parameters for LVEF in the multivariable models due to the limited sample size and the strong correlation between LVEF and GLS, which raised concerns about collinearity.

As expressed in the supplementary Table 2, participants were more likely to be excluded due to poor image quality if they had prevalent AF compared to the included group. Because AF is known to influence LA strain metrics, this divergence may affect the applicability of our findings to a HF population with more prevalent AF burden. Despite this, we have no reason to believe that there was any differential selection of the study subjects that would affect image quality subsequent strain analysis, suggesting minimal selection bias, see supplementary Table 2. We also note that bi-plane LA strain was not performed, as many patients had inadequate A2C views. Although bi-plane imaging allows for analysis of additional LA segments, there is currently no compelling evidence that it adds diagnostic value over four-chamber imaging alone [25]. There is a need to further validate the utility of using only the four-chamber view for LA strain which could potentially improve the feasibility [14]. During follow-up, 62 deaths were recorded, but only 22 were due to cardiac causes. This limited our ability to perform adequately powered analyses restricted to cardiac mortality, and we therefore used all-cause mortality as a more robust endpoint. Finally, NT-proBNP, LAVi and detailed diastolic assessment were not included in the multivariable models. These conventional markers are closely linked to LA strain and GLS and may introduce collinearity and over-adjustment, which could obscure the independent contribution of strain. The subjects included in HARVEST-Malmö were mainly of European descent, and the conclusions drawn might not be generalizable to all ancestries [26].

## Conclusions

In this prospective, long-term study of patients with acute heart failure, lower values of LAr and GLS predicted poorer outcomes. Lower LAr was associated with higher risk of both death and HF rehospitalization, and GLS was associated with higher mortality risk. These echocardiographic parameters should be further investigated for risk assessment of patients with heart failure to identify those at higher risk of rehospitalization and death.

## Electronic Supplementary Material

Below is the link to the electronic supplementary material.

Supplementary Table 1. Cox regression analyses for the association between LVEF with all-cause mortality and HF rehospitalization

Supplementary Table 2. Baseline Characteristics of Included and Excluded Participants

Supplementary Table 3. Causes of death during follow-up

## Data Availability

No datasets were generated or analysed during the current study.
