# Supplementary material for: Left ventricular and atrial strain and the risk of mortality and rehospitalization in heart failure
Source: Echo Res Pract. 2026 Feb 16;13:5. doi: 10.1186/s44156-026-00106-6 (PMC12908388; doi:10.1186/s44156-026-00106-6)
Supplement: Supplementary file 1 — Supplementary Table 1. Cox regression analyses for the association between LVEF with all-cause mortality and HF rehospitalization [file 44156_2026_106_MOESM1_ESM.docx]

**Supplementary material**

**Supplementary table 1.** Cox regression analyses for the association between LVEF with all-cause mortality and HF rehospitalization

|  | **All-cause mortality** | | **Heart failure rehospitalization** | |
| --- | --- | --- | --- | --- |
|  |  |  |  |  |
| ***Variables*** | **HR (CI 95%)** | **p-value** | **HR (CI 95%)** | **p-value** |
| ***Continuous LVEF*** |  |  |  |  |
| *Model 1* | 0.99 (0.98-1.01) | 0.123 | 0.98 (0.97-0.99) | **1.0 x 10^- 4^** |
| *Model 2* | 0.99 (0.98-1.01) | 0.153 | 0.98 (0.97-0.99) | **1.2 x 10^- 4^** |
| *Model 3* | 0.99 (0.98-1.01) | 0.283 | 0.98 (0.97-0.99) | **2.6 x 10^- 4^** |

Model 1: Adjusted for age and sex

Model 2: Adjusted for age, sex, moderate kidney disease, hypertension

Model 3: Adjusted for age, sex, moderate kidney disease, hypertension, fasting plasma glucose, current smoking, prevalent AF
