# Supplementary material for: Left ventricular and atrial strain and the risk of mortality and rehospitalization in heart failure
Source: Echo Res Pract. 2026 Feb 16;13:5. doi: 10.1186/s44156-026-00106-6 (PMC12908388; doi:10.1186/s44156-026-00106-6)
Supplement: Supplementary file 2 — Supplementary Table 2. Baseline Characteristics of Included and Excluded Participants [file 44156_2026_106_MOESM2_ESM.docx]

**Supplementary Table 2.** Baseline Characteristics of Included and Excluded Participants

| **Characteristics** | **Included (n=141)** | **Excluded (n=244)** | **p** |
| --- | --- | --- | --- |
| Age (years; (SD)) | 71 (13) | 74 (12) | 0.091 |
| Sex (male n;(%)) | 106 (75) | 165 (68) | 0.118 |
| Current smoking (n; (%)) | 18 (13) | 32 (13) | 0.910 |
| BMI (kg/m^2^; (SD)) | 27 (6) | 28 (6) | 0.055 |
| SBP (mmHg; (SD)) | 140 (31) | 138 (27) | 0.554 |
| DBP (mmHg; (SD)) | 81 (17) | 80 16) | 0.655 |
| Diabetes (n; (%)) | 49 (35) | 88 (36) | 0.748 |
| Prevalent AF (n; (%)) | 49 (35) | 125 (53) | **0.001** |
| Prior myocardial infarction | 50 (36) | 81 (34) | 0.719 |
| Moderate kidney disease (n; (%)) | 100 (71) | 183 (76) | 0.251 |
| eGFR (mL/min/1.73 m2; (SD)) | 48 (18) | 47 (18) | 0.570 |
| Hypertension (n; (%)) | 62 (44) | 108 (44) | 0.928 |
| Prior heart failure (n; (%)) | 86 (61) | 153 (60) | 0.733 |
| NTproBNP (pmol/L; (SD)) | 7092 (7507) | 6665 (7276) | 0.588 |
| Ejection fraction (%; (SD)) | 37 (15) | 39 (16) | 0.324 |
