# Supplementary material for: Left ventricular and atrial strain and the risk of mortality and rehospitalization in heart failure
Source: Echo Res Pract. 2026 Feb 16;13:5. doi: 10.1186/s44156-026-00106-6 (PMC12908388; doi:10.1186/s44156-026-00106-6)
Supplement: Supplementary file 3 — Supplementary Table 3. Causes of death during follow-up [file 44156_2026_106_MOESM3_ESM.docx]

**Supplementary Table 3.** Causes of death during follow-up

| **Cause of Death** | Total deaths, n =62 |
| --- | --- |
| Heart failure, n (%) | 15 (24) |
| Cardiac arrest, n (%) | 7 (11) |
| Stroke, n (%) | 1 (2) |
| Cancer, n (%) | 1 (2) |
| Other non-cardiovascular causes, n (%) | 38 (61) |
